# The First Matches

**Published:** 1889-07

**Authors:** 


### American Dental Association.

### The First Matches.

In a conversation a few days ago with the Hon. John D. Caldwell, of Cincinnati, the following interesting facts were brought out. Prior to the year 1833 all the friction matches used in America were imported, chiefly from England. In the year above mentioned Thompson & Hoge, a drug firm in Zanesville, Ohio, in their line of business imported friction matches from London. Bill Thompson, as the senior member of the firm was usually called, had his curiosity aroused as to the make-up of the matches, and as a result he made an analysis of the business end of the match—not the stick—which enabled him to prepare a similar material; this material was mixed and triturated in a mortar by Mr. Caldwell, above referred to, who was then a young man of all work in the drug-store, and thus it is, that we see who it was that helped to raise Lucifer [matches] in this country. The sticks were made by Mr. Jas. Steward. It may be that these facts are in the literature of the match, if the match has any literature, but they were new to us, as they probably will be to all match-scratchers, who go on firing away with them, neither knowing nor caring whence they came.

JUNE 25, 1889.

TO THE EDITOR OF THE DENTAL REGISTER,

*Dear Sir:*—Members of the American Dental Association, as well as others interested in the progress of the profession, are cordially requested to furnish contributions pertaining to dental education, literature and nomenclature, either in the form of papers or suggestions. Contributions should be in the hands of the officers of Section 2 on or before August 1, 1889.

Respectfully,

W. H. ATKINSON, Chairman,
41 E. 9th Street, New York.

LOUIS OTTOFY, Secretary, 70 Dearborn Street, Chicago.

NEWARK, N. J., June 3, 1889.

THE DENTAL REGISER,

The nineteenth annual session of the New Jersey State Dental Society will be held at the West End Hotel, Asbury Park, commencing Wednesday, July 17, and continuing 18 and 19 inclusive. Prominent dentists from throughout the country will read interesting papers, and the clinics will be more than usually instructive. Every thing new and useful in operative and mechanical dentistry will be exhibited by the inventors and dental supply houses for whom space will be reserved. Low hotel rates will prevail.

CHARLES A. MEEKER, D.D.S., Secretary.

